# Converting N_2_ molecules into NH_3_ with TiO_2_/Fe_3_O_4_ composite covered with a thin water layer under ambient condition

**DOI:** 10.1038/s41598-023-34685-9

**Published:** 2023-05-12

**Authors:** Ichiro Moriya

**Affiliations:** South wing 101, Maebara-nishi 3-6-3, Funabashi, Chiba Japan

**Keywords:** Catalysis, Materials science

## Abstract

As ammonia manufacture today require huge energy and very pure hydrogen gas and moreover emit large quantities of CO_2_, researches for new ammonia synthesis methods are actively performed. Here, author reports the novel method through which N_2_ molecules in air is reduced into ammonia with TiO_2_/Fe_3_O_4_ composite having thin water layer on composite’s surface under ambient condition (less than 100 °C and atmospheric pressure). The composites were composed of both nm-sized TiO_2_ particles and μm-sized Fe_3_O_4_ ones. First, composites were held in refrigerator, mainly at that time, N_2_ molecules in air adsorbed onto surface of composite. Next, the composite was irradiated with various lights including solar light, 365 nm LED light and tungsten light through thin water layer formed by condensation of water vapour in air. Reliable amount of ammonia was obtained under 5 min’s irradiation of solar light or of both 365 m LED light and 500 W tungsten light. This reaction was catalytic reaction promoted by photocatalytic one. In addition, holding in freezer instead of refrigerator provided larger amount of ammonia. Maximum ammonia yield was approximately 18.7 μmol/g 5 min under irradiation of 300 W tungsten light only.

## Introduction

Ammonia is essential chemical compound because almost all farm products depend on the ammonia-based fertilizers. Haber–Bosch method for artificial ammonia synthesis dominates ammonia producing field more than 100 years, however, it require large amount of energy in order to maintain the process [high temperature (350–525 °C) and high pressure(10-30Mpa)], and it also consumes large quantity of fossil fuel for acquiring hydrogen (H_2_) gas as a reactant. Moreover, it emits enormous volume of CO_2_ in hydrogen generation process^1^. In order to save energy and to decrease the CO_2_, many researches for alternative methods were performed, and continue as yet.

First, photocatalytic methods were researched in 1970s–1980s^2–7^. They were performed under mild condition (room temperature and atmospheric pressure) with high energy UV (ultra violet)-light irradiation, however, their yield was low, and, some report pointed out difficulty of reproducibility of ammonia yield reported^8^.

In recent years, researches for new energy-saving methods have been actively conducted. New methods and materials including electrochemical^9–17^, organometallic^18–21^ and new advancing materials^22–29^ were researched. Precious reviews with regard to their history and researches were informed^30–38^. More recent years, noteworthy works under ambient condition were reported. Ashida et al. reported efficient organometallic method using samarium (II) diiodide (SmI_2_) as reducing agent under ambient condition (room temperature and atmospheric pressure)^39^. Another work was performed by Hattori et al.^40^. They opened new way by using CaFH as electron donner under nearly room temperature (50 °C) and atmospheric pressure. These two reports showed clearly that ammonia synthesis under room temperature and atmospheric pressure without addition of outer energy like electric power was undoubtedly possible. These methods are steadily advancing for rearising next generation ammonia synthesis.

However, no reports in which ammonia has been synthesised by reducing N_2_ molecules in air with inexpensive catalyst under irradiation of solar light or various artificial lights and under ambient temperature and atmospheric pressure were founded.

The aim of this research is obtaining ammonia from N_2_ molecules in air with the original method under irradiation of various lights and under ambient condition.

Experiments were performed by two types of methods. One was experiments under irradiation of solar light, and, another method was one under irradiation of artificial light sources; both 365 nm Light Emitting Diode (LED) light and tungsten light or 365 nm LED light only or tungsten light only in a room.

Author reported the original method for converting CO_2_ into low-molecular-weight organic compounds with the TiO_2_/ZrO_2_ composites covered with a very thin layer of water under solar light irradiation in 2017^41^. Author calls the method WLOC (water layer over catalyst) method. Author’s method is good at reducing very stable molecules like CO_2_. In this method, TiO_2_ particles were used as photo-catalyst, and reaction is catalytic one assisted by TiO_2_ photo-catalyst. Characteristics of author’s method are using catalyst covered with very thin water layer, and being conducted in gas phase. This time, the WLOC method was applied to generate ammonia from N_2_ molecules in air with TiO_2_/Fe_3_O_4_ composites under irradiation of various lights and under ambient condition. Successful results under ambient conditions for both temperature and pressure were acquired.

In relation to this research, Fujishima et al. reported the photo-catalyst effect of TiO_2_^42^. Specifically, irradiation with UV light generates electrons (e^−^) and holes (h^+^) in TiO_2_. The electrons provide the reduction driving force, and the holes provide the oxidation driving force. Later, Sato et al. reported the generation of H_2_ and O_2_ using wet Pt/TiO_2_ under irradiation by light with energy equal to the band-gap energy of TiO_2 (_1980) ^43,44^. Moreover, they demonstrated that H_2_ and O_2_ were generated by the splitting of H_2_O added.

In this research, the photocatalytic effect played important role for promoting ammonia generation and wet condition reported by Sato et al. also was essential for the WLOC method.

In this report, items below were studied.

First: Ascertaining whether the thin water layer on the surface of composites is effective.

Second: Study on preparing method of TiO_2_/Fe_3_O_4_ composite. (on importance of interface made up by TiO_2_ particles and Fe_3_O_4_ ones).

Third: Desirable experimental procedures for composites before irradiation of light.

Forth: Dependence of ammonia yield on TiO_2_/Fe_3_O_4_ ratios. (TiO_2_/Fe_3_O_4_ ratios were changed between 10/0 and 0/10).

Fifth: Dependence of quantity of ammonia generated on temperature of atmosphere (average temperature during irradiation of light) in bag.

Sixth: Experiments using Argon gas as atmosphere.

Seventh: Increasing quantity of ammonia generated

### Experiments

The author devised composites consisting of nanometre-sized TiO_2_ particles and micrometre-sized magnetite (Fe_3_O_4_) particles and applied them to author’s original method (WLOC method). First, particles or molecules adsorbed onto the composite’s surface were removed by the elimination of static electricity using an ionizer (Kasuga Denki Inc Model KD-150W). Next, after the composites were cooled in a refrigerator for more than 24 h (desirably 48 h), they were placed into a transparent gas-barrier plastic bag with high-temperature (approximately 30 °C) and high-humidity (60–90%) room air.

Cooling in a refrigerator enabled the whole surface of the composite to be covered with a very thin layer of water via the rapid condensation of water vapour in the air after taking out of the composite from refrigerator to reproduce Sato’s condition (wet condition). This method is unique because the reduction of N_2_ molecules is performed in air (or more precisely, in the air phase via a very thin water layer). Moreover, this method increases the efficiency of the light irradiation because real solar light or artificial lights directly irradiated onto the composites.

In this study, the TiO_2_/Fe_3_O_4_ composites increased the amount of generated ammonia. The composite exhibited morphology that Fe_3_O_4_ particles were covered by TiO_2_ ones. The new composites are composed of two types of inorganic compounds (a, b) that were combined and pressed together. The first inorganic compound (a) is characterized by nanometre-sized anatase TiO_2_ photo-catalyst particles, and the other inorganic compound (b) is composed of micrometre-sized Fe_3_O_4_ particles. The weight ratio (TiO_2_/Fe_3_O_4_) of the composites is mainly 1:1. The use of such a large amount of additive (Fe_3_O_4_) is unique. After (a) and (b) were pressed to form the composites (c), the composites (c) were scattered onto an electric conducting material such as a copper plate. The copper plate can facilitate the transfer of electrons and holes from one composite to the other. Then, the composites and Cu plate were held in a glass laboratory dish (the glass laboratory dish containing the composites and Cu plate is hereafter referred to as the test unit).

**Figure1:**
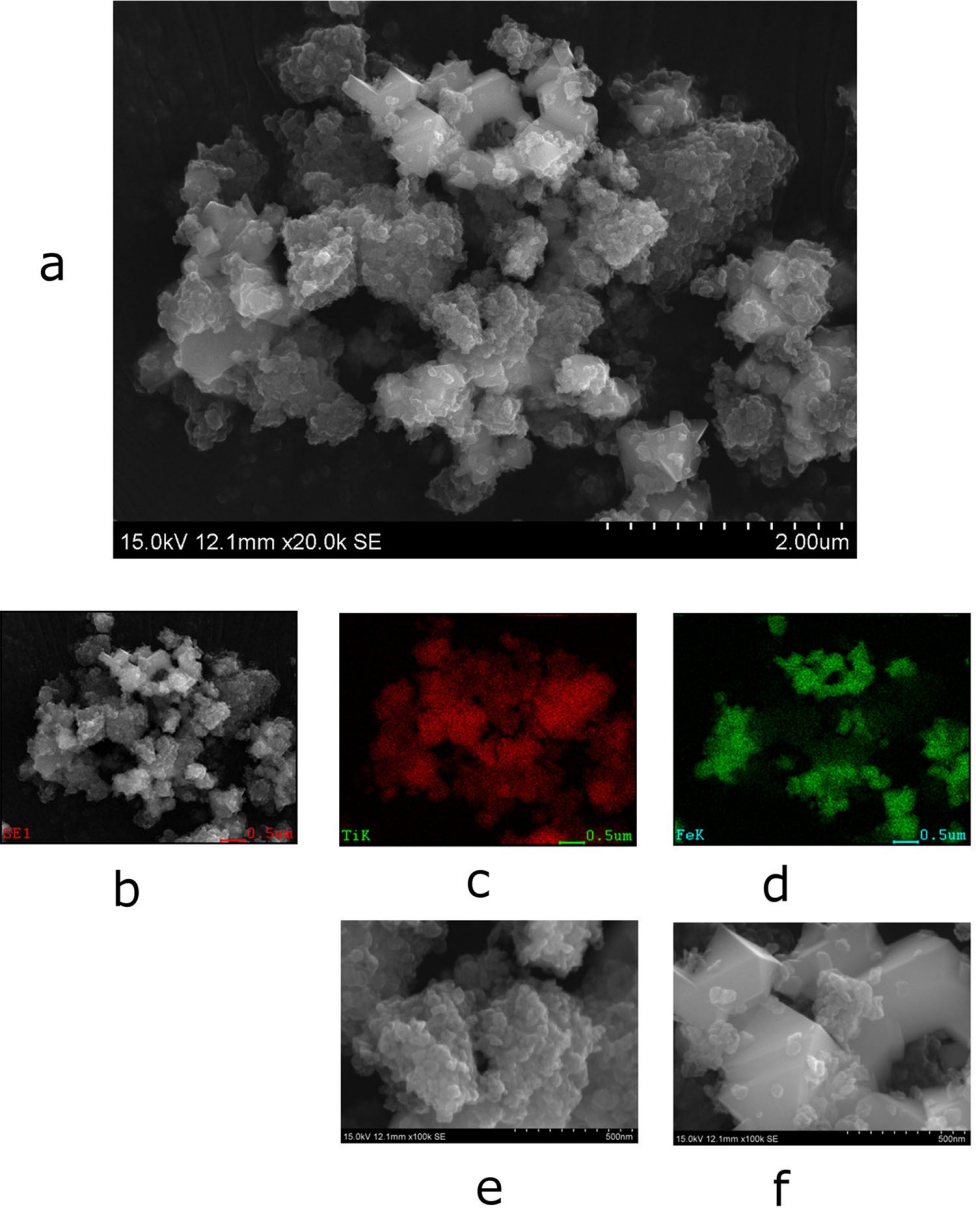
Magnification SEM image of the composite. (**a**) Low-magnification SEM image of the composite. This image is a low-magnification (×20,000) micrograph of typical composites. (**b**) micrograph that is equal to image (**a**) (normal image). (**c**) Ti atom’s distribution (correspond to ash colour part of micrograph (**a,b**)). (**d**) Fe atom’s distribution (correspond to white part of micrograph (**a,b**)). (**e**) High-magnification (×100,000) micrograph show a part of Ti atoms in micrograph (**c,f**) High- magnification (×100,000) micrograph show a part of Fe atoms in micrograph d. Sample was one used in “Result” section. (**c**) “Desirable experimental procedures before irradiation of lights” part, and times of use was equal to 1.

Figure 1 shows SEM image of the composite and distribution of Ti atoms or Fe ones with water vapour condensed on its surface and with the thin water layer evaporated away (SEM: Hitachi High Technology’s Co. Ltd., SU6600). Figure 1a shows that numerous nanometre-sized TiO_2_ particles are present on the top layer of the composites, and the core or inner part of the composite is composed of micrometre-sized Fe_3_O_4_ particle. From atom’s distribution (Fig. 1c, d), numerous nanometre- sized particles are TiO_2_ and larger μm-sized particles are Fe_3_O_4_. Figure 1e shows a higher-magnification (100,000×) SEM image correspond to part that Ti atoms detected in Fig. 1c. Many TiO_2_ particles exist at the surface. Figure 1f shows a same-magnification (100,000×) SEM image of part that Fe atoms detected in Fig. 1d. Angular crystals of Fe_3_O_4_ expose on surface.

**Figure2:**
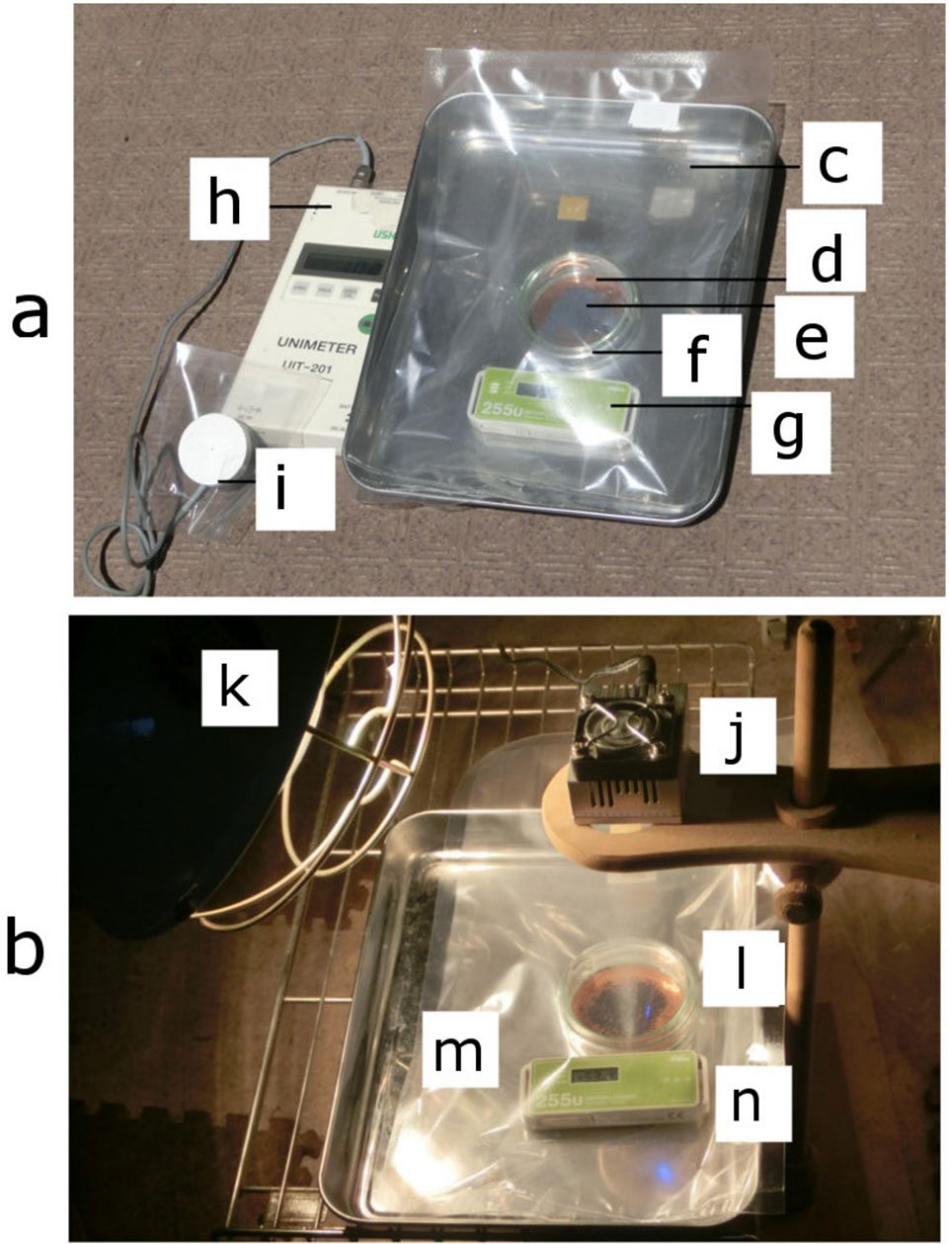
Photograph taken during experiments. (**a**) Experiment used solar light. (**c**) Gas-barrier plastic bag; (**d**) Cu plate, (**e**) composites (TiO_2_:Fe_3_O_4_ = 1:1), (**f**) test unit, (**g**) thermometer, (**h**) solar-energy-detecting instrument (UNIMETER), (**i**) solar-energy-detecting sensor. (**b**) Experiment used artificial lights. (**j**) 365 nm LED light, (**k**) 300W tungsten light, (**l**) test unit, (**m**) gas-barrier plastic bag, (**n**) thermometer.

Figure 2 shows photographs of the experiments under irradiation of two kinds of light source. One is the experiment under irradiation of real solar light (Fig. 2a), another is the one under irradiation of both 365 nm LED light and 300W tungsten light in a room (Fig. 2b).

Solar light experiments and artificial light ones were mainly performed during the Japanese summer season, which provides high temperatures and high humidity. During irradiation, the test unit was placed into a transparent gas-barrier plastic bag with 1000 ml of air. The bag that transfers the light and contains the produced ammonia was used. The bag allowed both the concentration of ammonia and the inner volume to be measured after irradiation of solar light or artificial lights. Light illumination started 6–7 min after taking out from refrigerator (internal temperature: 1–4 °C, humidity: 35–45%). After irradiation, the bag was cooled to a temperature inside the bag of 35 °C. or less, then concentration of ammonia in the bag was measured using gas-detecting tubes.

A gas-barrier plastic bag through which oxygen (O_2_) or nitrogen (N_2_) molecules cannot penetrate was used.

The experiments were conducted in Funabashi city in the Chiba prefecture of Japan (lat. 35°70′ N and long. 140°02′ E).

## Result

In the subsequent description, the term “dry conditions” refers to experiments in which the test unit was not cooled in a refrigerator, and no formation of thin water layer on the composites was generated. Whereas the term “wet conditions” refers to experiments in which the test unit was cooled in a refrigerator, resulting in the formation of a thin water layer on the composites.

Table 1 show the experimental conditions, and, Table 2 show the experimental results, respectively. Table 1 and Table 2 make a pair.

**Table 1:** Experimental conditions.

Group	No	Date	Sample composition	Injection gas	Irradiation time	Weather	Irradiation^a^ intensity	Sample condition
			TiO_2_/Fe_3_O_4_				mW/cm^2^	
A	1	2021/7/21	1/1	Air	12:10–12:15 (5 min)	Clear	1.1	Wet
	2	2021/7/22	1/1	Air	11:45–10:50 (5 min)	Clear	0.9	Wet
	3	2021/8/20	1/1	Air	12:30–12:35 (5 min)	Clear	1.2	**Dry**
	4	2021/7/22	1/1	Air	12:15–12:25 (10 min)	Clear	0.9	Wet
	5	2021/8/1	1/1	**N2**	11:35–11:40 (5 min)	Clear	1.0	Wet
	6	2021/8/1	1/1	**N2**	11:00–11:10 (10 min)	Clear	1.0	Wet
	7	2021/8/4	**None**	Air	11:50–11:55 (5 min)	Clear	1.2	–

^a^Irradiation intensity was measured at 365 nm in wavelength.

Significant values are in bold.

**Table 2:** Experimental results-1.

Group	No	Holding in refrigerator (h)	Injector air (room air)		Irradiation time, t (min)	Temperature in bag		Product (t) Ammonia		Inner gas volume^a^ (ml)
			Temperature (°C)	Humidity (%)		Average (°C)	Range (°C)	ppm	μmol/ g t	
A	1	38	29.2	76	5	41.4	29.1–51.3	10	1.92	1000
	2	22	29.3	75	5	40.3	29.0–49.1	10	1.92	1000
	3	**0**	28.6	76	5	41.4	28.5–51.3	3	0.58	1000
	4	17	29.5	73	**10**	53.3	29.0–62.3	1	0.19	1000
	5	21	(29.6)	(74)	5	40.7	28.7–49.7	10	1.92	1000
	6	37	(29.2)	(73)	**10**	47.8	28.3–60.3	10	0.96	1000
	7	24	29.7	73	5	41.9	29.3–50.9	–	–	1000

Samples of 0.2 g were irradiated with only real solar light. And, sign “−” shows that color change of gas-detecting-tube was not detected (reduced product is equal to almost 0).

^a^Volume of inner gas (after irradiation): there is inaccuracy from − 20 ml to + 10 ml.

Significant values or noticeable conditions are in bold.

### I. Experiments using solar light

- Group A of Table 1 and 2
  Experimental No. 1 and No. 2 show that N_2_ molecules in air were reduced into ammonia with TiO_2_/Fe_3_O_4_ (1/1) composites under ambient condition and under irradiation of solar light. Ammonia was clearly generated under irradiation of 5 min on wet condition. Experimental No. 3 shows that ammonia yield was very small on dry condition, that is wet condition was effective.
  Experimental No. 4 shows the case in which irradiation time was extended to 10 min. Ammonia yield was largely decreased. This result suggested that ammonia generated was oxidized and disappeared by photocatalytic oxidation effect of TiO_2_ component between 5 and 10 min. of irradiation. In order to ascertain above supposition, experiments (experimental No. 5 and No. 6) were performed under N_2_ atmosphere. As N_2_ is non-active molecule, it does not oxidize ammonia synthesised. Thus, ammonia is considered not to disappear after 10 min. of irradiation in N_2_ atmosphere.
  Experimental No. 5 showed that ammonia was synthesised during irradiation of 5 min., and concentrations of ammonia (ppm) of experimental No. 5 and No. 6 were same, thus ammonia generated was not decreased between 5 and 10 min. of irradiation in N_2_ atmosphere. That is, it was ascertained that in air atmosphere, ammonia was largely decreased by the photocatalytic oxidation effect of TiO_2_ component with O_2_ molecules in air between 5 and 10 min of irradiation. In author’s experiments conducted in gas phase using air in the past, photocatalytic oxidation reaction of TiO_2_ occurred in dry condition and photocatalytic reduction one of TiO_2_ occurred in only wet condition. Therefore, these results also showed that thin water layer over the composites disappeared by increasing of composite’s temperature under long time of irradiation to form dry condition, and photocatalytic effect of TiO_2_ component changed from reducing effect to oxidation one.
  That is, water layer over composite (wet condition) was essential for protecting photocatalytic oxidation reaction.
- Blank test
  Brank tests (experimental No. 7) was cases of no TiO_2_/Fe_3_O_4_ (1/1) composites, and showed that no ammonia was detected under irradiation of solar light. Thus, there were not elements generating ammonia except for TiO_2_/Fe_3_O_4_ composites.

### II. Experiments using artificial lights (Group B–L)

In these sections, the term” sample” refers to as TiO_2_/Fe_3_O_4_ composite.

Continuously, two kinds of artificial light sources (365 nm LED light and 300W tungsten light) were used instead of solar light. By the use of both 365 nm LED light and tungsten light, their combined wavelength-area could substitute that of solar light. 365 nm LED light only and 300W tungsten light only were also used. Characteristic results were summarised as four kinds of graphs in Fig. 3.

(c) Desirable experimental procedures before irradiation of lights
  **Figure3:**
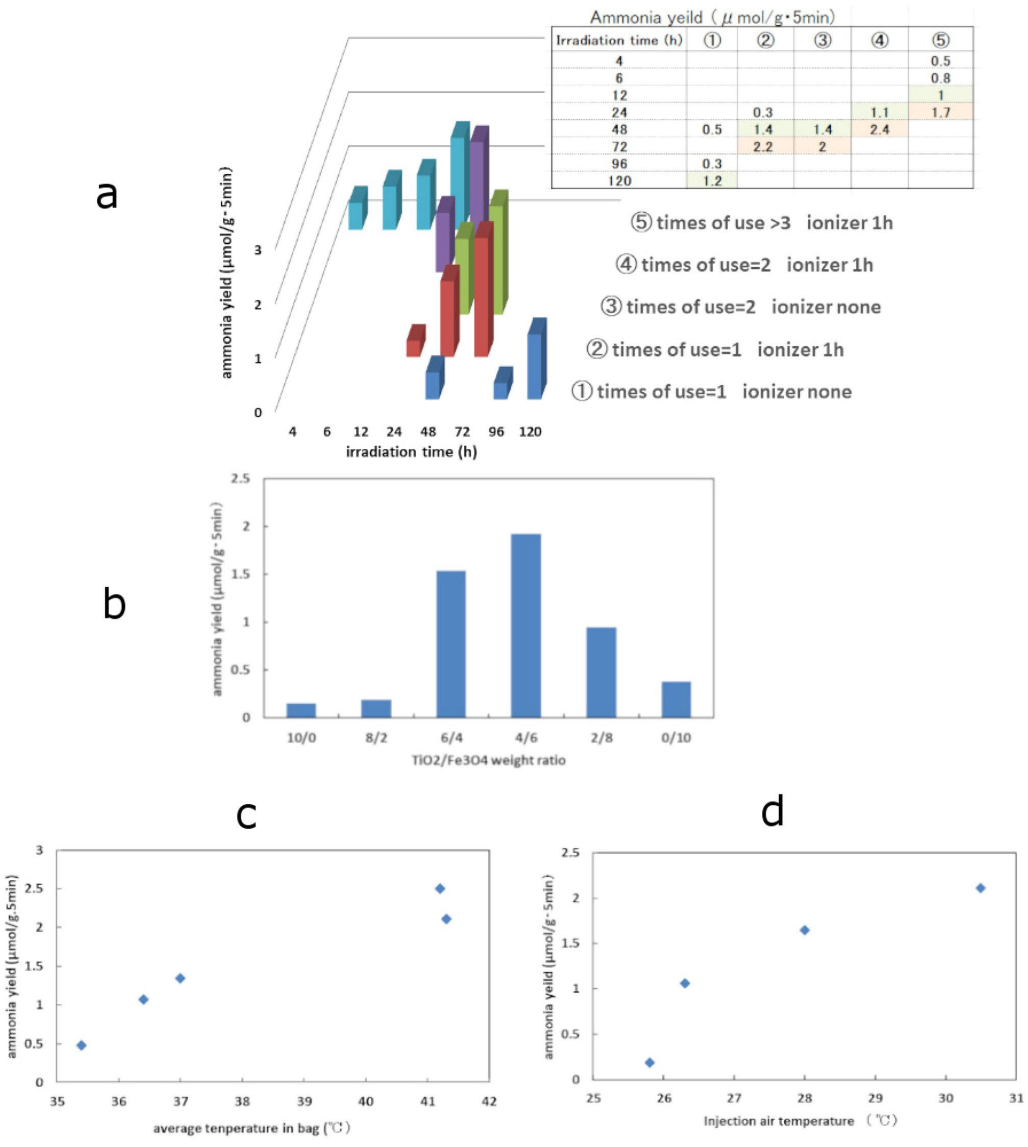
Ammonia yield as a function of variety of conditions. (**a**) Plots of ammonia yield as a function of holding time in refrigerator with various conditions of two factors (“use of ioniser” and ”times of use”). (**b**) Plots of ammonia yield as a function of TiO_2_/Fe_3_O_4_ ratio (10/0–0/10). Plots in this figure correspond to the results in the Group D of Table 3. (**c**) Plots of ammonia yield as a function of average temperature in bag during irradiation of lights. Plots in this figure correspond to the results in the Group E of Table 3. (**d**) Plots of ammonia yield as a function of injection air temperature (injection air is equal to room air). Plots correspond to the results in the Group F of Table 4.
  In order to obtain larger ammonia yield and to acquire accurate values of ammonia generated, desirable experimental procedures were evaluated. Figure 3a shows effect of three factors including “removing of static-electricity by ionizer”, “times of use (times that a sample was used in experiments)” and “holding time in refrigerator”. All factors increased ammonia synthesised. Figure 3a shows ammonia yield as function of irradiation time with variety of conditions. The results were following.
  Removing static electricity by ionizer decreased holding time in refrigerator to offer larger ammonia yield stably. (Decreasing of holding time is desirable.)
  Required holding time in refrigerator decreased with increasing of times of use.
  Without removing of static electricity, with first use after preparation of composites, it was necessary to hold very long time (more than 120 h) in refrigerator.
  That is, desirable experimental procedure contained following three items.
  1. Removing static electricity by ionizer: 1 h
  2. Times of use: more than 3 times
  3. Holding time in refrigerator: more than 24 h (desirably 48 h)
(d) Group B of Table 3: Blank test

**Table 3:** Experimental results-2.

Group No.	Sample composition (TiO_2_/Fe_3_O_4_)	Times of use	Ionizer (h)	In refrigerator (h)	Injection air (room air)		Irradiation			Temperature in bag		Product		Volume of air^a^ (ml)
							Source^b^	Time	Intensity (mV/cm^2^)					
					Temperature (°C)	Humidity (%)				Average	Range	Ammonia		
												ppm	μmol g 5 min	
B 8	Bag only	–	–	–	25.7	73	LED& T	5	1.5	44.5	26.4–56.8	0.7	0.13	1000
9	Without sample	–	1	49	25.5	71	LED& T	5	1.5	44.1	24.3–59.3	1	0.19	1000
C 10	1/1	6	1	48	30.5	70	LED& T	5	1.5	43.2	29.2–55.0	11	2.11	1000
11	1/1	2	1	48	30.2	71	LED& T	5	1.5	45.6	29.1–58.8	12.5	2.40	1000
12	**1 + 1**^c^	1	1	29	28.8	72	LED& T	5	1.5	38.2	28.1–47.3	1.5	0.29	1000
13	**1 + 1**^c^	2	1	23	29.7	75	LED& T	5	1.5	39.8	29.4–49.1	4.5	0.86	1000
D 14	**TiO**_**2**_** only**	1	1	47	27.8	80	LED& T	5	15	38.3	27.7–47.1	0.8	0.15	1000
15	**8/2**	1	1	15	30.0	74	LED& T	5	1.5	38.1	29.9–45.6	1	0.19	1000
16	**6/4**	3	1	24	30.4	71	LED& T	5	1.5	43.6	29.2–53.9	8	1.54	1000
17	**4/6**	5	1	27	30.2	72	LED& T	5	1.5	45.3	29.6–58.8	10	1.92	1000
18	**2/8**	1	1	26	29.4	72	LED& T	5	1.5	44.0	28.8–55.4	5	0.95	1000
19	**Fe**_**3**_**O**_**4**_** only**	1	1	23	29.2	73	LED& T	5	1.5	38.5	28.4–47.2	2	0.38	1000
E 20	1/1	9	1	72	30.1	75	LED& T	5	1.5	**35.4**	29.4–40.2	2.5	0.48	1000
21	1/1	9	1	72	30.0	70	LED& T	5	1.5	**36.4**	29.5–42.1	5.5	1.06	1000
22	1/1	29	1	48	30.1	73	LED& T	5	1.5	**37.0**	29.5–43.4	7	1.34	1000
23	1/1	3	1	94	30.0	68	LED& T	5	1.5	**41.2**	29.5–51.1	13	2.50	1000
24	1/1	10	1	48	30.7	71	LED& T	5	1.5	**41.3**	31.0–50.0	11	2.11	1000
F25	1/1	6	1	48	**30.5**	70	LED& T	5	1.5	43.2	29.2–55.8	11	2.11	1000
26	1/1	7	1	89	**28.0**	68	LED& T	5	1.5	48.9	27.5–67.6	8.5	1.63	1000
27	1/1	21	1	95	**26.3**	73	LED& T	5	1.5	49.5	25.5–65.3	5.5	1.06	1000
28	1/1	4	1	48	**25.8**	75	LED& T	5	1.5	47.9	25.5–67.5	1	0.19	1000

Samples of 0.2 g were irradiated with both 365 nm LED light and 300 W tungsten light.

^a^There is inaccuracy from −20 ml to + 10 ml.

^b^The term ”LED&T” refers to as 365 nm LED light and tungsten light.

^c^Sample was prepared with mixing only without pressing.

Significant values or noticeable conditions are in bold.
  Group B of Table 3 shows the results of blank tests in which ammonia was very slightly detected under various conditions without sample (TiO_2_/Fe_3_O_4_ composite) under irradiation of both 365 nm LED light and 300W tungsten light. Experimental No.8 shows that ammonia was also slightly detected from gas barrier plastic bag containing air of 1000 ml. Thus, the bag was considered to adsorb small amount of ammonia. Before this experiment, ammonia also was slightly detected in room air (0.5 ppm). As ammonia exists in homes of Japan in summer season (average: 38 μg/m^3^, maximum: 1000 μg/m^3^)^45^, this value measured in summer season (0.5 ppm, 380 μg/m^3^) was reasonable one. Experimental No. 9 shows that ammonia was detected from test unite without sample. The detected value was also considered to be that of ammonia absorbed on surface of bag and test unite, that is, inner surface of bag, and on surface of glass laboratory dish and Cu plate.

### Group C–H

Group C–H show experimental results performed using artificial light sources, thus three kinds of light resources including “both 365 m LED light and 300 W tungsten light”, “365 m LED light only”, “300W tungsten light only” were used instead of solar light.

Variety of conditions were examined, and the results were summarized in Tables 3 and 4.

(e) Group C of Table 3
  **Table 4:** Experimental results-3.

Group	No	Sample composition (TiO_2_/Fe_3_O_4_)	Times of use	Ionizer (h)	In refrigerator (h)	Injection air (room air)		Irradiation			Temperature in bag		Product		Volume of air^a^ (ml)
								Light source^b^	Time (min)	Intensity ( mV/cm^2^)					
						Temperature (°C)	Humidity (%)				Average	Range	Ammonia		
													ppm	μmol/ g 5 min	
G	29	1/1	5	1	93	29.2	68	**LED only**	5	1.5	28.6	28.5–28.7	1.5	0.29	1000
	30	1/1	8	1	32	29.1	71	**LED only**	5	1.5	28.5	28.5–28.6	1.2	0.23	1000
	31	1/1	9	1	100	28.9	76	**T only**	5	0.08	37.0	28.8–44.9	7	1.34	1000
	32	1/1	4	1	48	29.3	77	**T only**	5	0.08	39.4	29.8–48.2	5	0.96	1000
H	33	1/1	10	1	48	Injection gas : **Ar**		LED& T	5	1.5	41.0	28.5–50.8	11	2.11	Argon1000
			11	–	11	Injection gas : **Ar**		LED& T	5	1.5	34.3	29.7–50.6	10.5	2.02	Argon900
			12	–	12	Injection gas :**Ar**		LED& T	5	1.5	37.8	29.5–54.2	10.5	2.02	Argon800
					In freezer (h), (°C)										
I	34	1/1	8	2	164, -13	26.2	78	T only	5	0.08	34.7	25.2–44.1	90	18.7	1000
	35	1/1	10	1	49, -18	26.2	75	T only	5	0.08	38.7	25.1–49.5	75	15.6	1000
	36	1/1	8	1	106, -20	26.0	79	T only	5	0.08	38.1	25.5–48.7	65	13.5	1000
	37	1/1	20	1	71, -23	25.0	75	T only	5	0.08	47.2	24.2–66.4	55	11.4	1000
J	38	TiO_2_ only	2	1	47, -17	28.1	78	T only	5	0.08	37.9	26.9–48.1	2.5	0.48	1000
	39	Fe_3_O_4_ only	1	1	45, -19	26.3	79	T only	5	0.08	37.0	26.0–46.9	6.3	1.21	1000
K	40	**ZrO**_**2**_**Ep/Fe**_**3**_**O**_**4**_** (1/1)**		1	46, -18	26.5	78	T only	5	0.08	37.3	25.4–47.7	39	8.10	1000
	41	**ZrO**_**2**_**3N/Fe**_**3**_**O**_**4**_** (1/1)**		1	48, -15	26.1	77	T only	5	0.08	38.3	26.3–46.3	7	1.34	1000

Samples of 0.2 g were irradiated with both 365 nm LED light and 300 W tungsten light or 365 nm LED light only or 300 W tungsten light only.

^a^There is inaccuracy from −20 ml to + 10 ml.

^b^The term “LED” refers to as 365 nm LED light, and the term ”T” refers to as tungsten light.

Significant values or noticeable conditions are in bold.
  On the amount of ammonia synthesised, very large deference was showed between No. 10–11 and No. 12–13. In No. 10–11 composites were prepared with strong press during mixing, whereas, in No. 12–13, samples were prepared with no press and only mixing.
  TiO_2_/Fe_3_O_4_ (1/1) composites with both mixing and pressing provided larger ammonia yield than one with mixing only. Thus, it was indicated that preparing method with both mixing and pressing was very effective.
  On the surface of composites with press during mixing, large amount of interface between TiO_2_ particles and Fe_3_O_4_ ones are formed, whereas, in the case of no press, the interface exists at only contact point between TiO_2_ particles and Fe_3_O_4_ ones. Therefore, possible explanation is that reaction cites at which ammonia is synthesised exist at the interface. Incidentally, standard method of this report is strong press during mixing.
  (f) Group D of Table 3
    In experimental No. 14–19, weight ratio of TiO_2_ component and Fe_3_O_4_ component were changed (10/0–0/10). Figure 3b shows relationship between TiO_2_/Fe_3_O_4_ ratio and ammonia yield.
  It showed volcano-plot and maximum ammonia yield was obtained at the ratio of nearly equal 1/1. In left side of top, ammonia yield increased with increasing of Fe_3_O_4_ component, in right side, ammonia yield increased with increasing of TiO_2_ component. As amount of interface of TiO_2_ particles and Fe_3_O_4_ ones also have same characteristic (volcano-plot), ammonia yield reached maximum at the point that interface was formed with maximum amount.
  (g) Group E of Table 3
  Average temperature in bag during irradiation of both 365 m LED light and tungsten light was changed. Data (experimental No.20–24) were summarized in Section E of Table 3. Figure 3c shows the ammonia yield as function of average temperature in bag during irradiation of both 365 nm LED light and tungsten light. Average temperatures were changed by changing energy power supplied to tungsten light by use of voltage adjustment device (Tokyo Glass Instrument Co., Led. Electro-Slider).
  Ammonia yield increased with increasing of the average temperature. This result indicated that reaction was just catalytic.
  (h) Group F of Table 3
  Experimental No. 25–No. 28 show the cases that temperature of injection air was changed under nearly equal humidity and under nearly equal average temperature in bag.
  Figure 3d shows the ammonia yield as function of temperature of injection air under nearly equal humidity. Higher temperature provided large ammonia yield; ammonia yield increased with increasing of water-layer-thickness over the composite, because higher temperature increase quantities of water vapour in air under nearly equal humidity, and lead water-layer-thickness over the composites to increase by condensation of the water vapour. On the contrary, as more thin water layer easily evaporate and disappear under irradiation of lights, consequently photocatalytic effect changes from reduction effect to oxidation one, then generated ammonia decrease.
  Enough-thickness-water-layer was essential for increasing ammonia yield.
  (i) Group G of Table 4, Experiments using single light source
  Experimental No. 29–32 show the ammonia yield in the case using only 365 nm LED light or only tungsten light.
  Irradiation of only the LED light hardly generated ammonia; photocatalytic effect of TiO_2_ component did not work for ammonia generation.
  And irradiation of only tungsten light unexpectedly provided considerable ammonia yield.
  (j) Group H of Table 4
  Next experiment (Experimental No. 33) is described in detail, because the experiments provided interesting information. End of experiments holding in refrigerator was ones using Ar gas as atmosphere in bag. On experimental No. 33, same sample and same gas in bag were continuously used, that is, TiO_2_/Fe_3_O_4_ (1/1) sample in bag was not changed and gas in bag was also not changed. Except for sampling the gas (100 ml) for measurements of ammonia concentration, the bag containing test unit and Ar gas was kept starting shape until end of experiment. During hold in refrigerator in second and third runs, original Ar gas was kept in the bag. The experiment consisted of three runs, and each run included holding in refrigerator, irradiation of lights and measurement of ammonia concentration. In first run, 10.5 ppm of ammonia was measured, and in second and third runs, approximately same values (10 ppm) were measured, thus ammonia concentrations were maintained. This result is considered to be following.
  1. In the first run, N_2_ molecules in air adsorbed on the surface of composite before injecting Ar gas, because atmosphere in bag was only Ar gas after injection of Ar gas.
  2. Adsorbing N_2_ molecules on surface of composites was reduced under irradiation of lights and ammonia was generated (11 ppm).
  3. In second and third run, ammonia concentrations (10.5 ppm) were nearly equal to the value (11 ppm) in first run; increasing or decreasing of ammonia concentration was not detected, that is, no ammonia generation under Ar gas atmosphere was produced in second and third runs.
  These results lead author to hit upon the suggestion that adsorbing N_2_ molecules onto surface of composites before injection of Ar gas into the bag was essential. And author immediately performed experiments holding in freezer (approximately −20 ℃) in place of refrigerator (1–5 ℃) in order to increase quantities of adsorbing N_2_ molecules,
  The reason is described in discussion part.

### III. Group I–K of Table 4

Experimental No. 34–No. 41 show experimental results performed with holding in freezer. Best conditions for the WLOC method are using solar light or both 365 m LED light and tungsten light as light source in summer season. However, summer season offered best condition (high temperature and humidity) have past, thus it was difficult to obtain enough thickness of water layer over composites. Irradiation of both 365 nm LED light and tungsten light was considered to decrease ammonia yield through photocatalytic oxidize effect of TiO_2_ component generated by irradiation of 365 nm LED light under dry condition after disappearing of water layer. Therefore, irradiation of tungsten light only was adopted.

(k) Group I of Table 4
  Experimental No. 34–37 show experimental results that TiO_2_/Fe_3_O_4_ (1/1) composite was examined under irradiation of only tungsten light after holding in freezer. They showed that larger amount of ammonia (12-19 μmol/g 5 min) than values obtained by holding in refrigerator (1.0–1.3 μmol/g 5 min in experimental No. 10–11) were detected.
(l) Group J of Table 4
  Experimental No. 38–39 show results of experiments in which TiO_2_ only or Fe_3_O_4_ only as catalyst was used. In combination of Fe_3_O_4_ catalyst and tungsten light, detected value was larger than that in combination of TiO_2_ catalyst and tungsten light, however, the value itself was very small than that of combination of TiO_2_/Fe_3_O_4_ (1/1) composite and tungsten light (experimental No. 34–37).
  (m) Group K of Table 4
  Final experiments (experimental No. 40–41) were those used ZrO_2_ in place of TiO_2_. ZrO_2_ having two kinds of particle size were used in order to ascertain advantage of fine-grained particle. ZrO_2_Ep (particle size: 6 nm) and ZrO_2_3N (particle size: 10 μm) were used. The results indicated that fine grained particle offered very large ammonia yield.

### IV. Composite’s properties and the surface temperature of composites under irradiation of tungsten lamp

1. Specific surface area of TiO_2_/Fe_3_O_4_(1/1) composite
  The specific surface area of the composite was 43 m^2^/g before use for experiments and 42 m^2^/g after used in 46 times of experiments. No reduction in specific surface area by experiments was observed. These indicate that the composite can be used repeatedly.
  As mentioned so far, no yield decline was observed when the same sample was actually used in many experiments.
2. The particle size distribution of the TiO_2_/Fe_3_O_4_(1/1) composite was measured by the laser method (wet method).
  Measuring device: HORIBA Laser Scattering Particle Size Distribution Analyzer Partica LA-950V2
  Type of particle size distribution: Clean single peak
  Median size(D50): 1.50098 (μm)
  Mean size: 1.95946 (μm)
  Mode size (the largest frequency of particles): 1.2282 (μm).
  $$\begin{aligned} {\text{Diameter on cumulative }}\% & { 1}0.00 \, \left( \% \right):0.{8}0{37 }(\mu {\text{m}}) \\ & {9}0.00\left( \% \right):{ 3}.{6339}(\mu {\text{m}}). \\ \end{aligned}$$
3. Comparison between the near composite temperature and the composite surface temperature during tungsten lamp irradiation.
  Measurement of composite surface temperature with a radiation thermometer during irradiation experiments cannot be performed because the gas barrier bag is in the way.
  Therefore, a comparison of the two was made without a gas barrier bag. Temperature near the composite was also measured without a gas barrier bag by ordinary thermometer that was used in all experiments in this report. For 3–5 min of irradiation, the surface temperature was approximately 20 °C higher than the near composite temperature. Since the TiO_2_/Fe_3_O_4_(1/1) composite is black, it absorbed rays of tungsten light and raised the surface temperature. In contrast, the surface temperature of the white TiO_2_ particles was exactly the same as the near composite temperature.
  “Temperature in bag” in the Tables 1, 2, 3 and 4 is the near composite temperature in gas barrier bag that was measured with an ordinary thermometer.

## Discussion

The solar light that reaches ground is larger than 300 nm in wavelength. The TiO_2_ photo-catalyst absorbs light less than 380 nm in wavelength. Therefore, the available wavelength range is only 300–380 nm. The energy of the light in this range is estimated to be approximately 3–4% of the entire solar energy.

However, solar energy of 3–4% is useful. Both e^-^ and h^+^ are generated by the TiO_2_ photo-catalyst.

In this report, the author used only TiO_2_, not Pt/TiO_2_. Therefore, the photocatalytic reaction schemes are based on the report of Fujishima et al.^42^:

$${\text{TiO}}_{{2}} + {\text{ 2 photons }}({\text{h}}\nu ) \to {\text{2 e}}^{ - } + {\text{2 h}}^{ + }$$

At oxidation sites on the TiO_2_ surface: H_2_O + 2 h^+^  → 1/2 O_2_ + 2H^+^

At reduction sites on the TiO_2_ surface: 2 e^−^ generate the reduction driving force.

H^+^ and e^−^ can be used to reducing reaction of N_2_ molecules. As the reduction driving force (−0.52 eV with respect to SHE^46^) is stronger than that of normal electrons, and, high energy e^-^ probably have advantage to reduction of N_2_ molecules.

As Fe based catalyst^47,48^ or fused-iron catalyst derived from magnetite (Fe_3_O_4_)^37^ is known to be catalyst that generates ammonia from N_2_ molecules with electron donner in Haver–Bosch method. And Fe_3_O_4_ component of TiO_2_/Fe_3_O_4_ (1/1) composite absorbs lays of visible and IR (infra-red) wavelength in solar light to become higher-temperature itself. Increasing temperature of Fe_3_O_4_ itself increases ammonia yield through catalytic reaction. While, TiO_2_ component is known to be photo-catalyst that absorbs wavelength less than 380 nm and produces photocatalytic effect that can supply many electrons.

**Figure 4:**
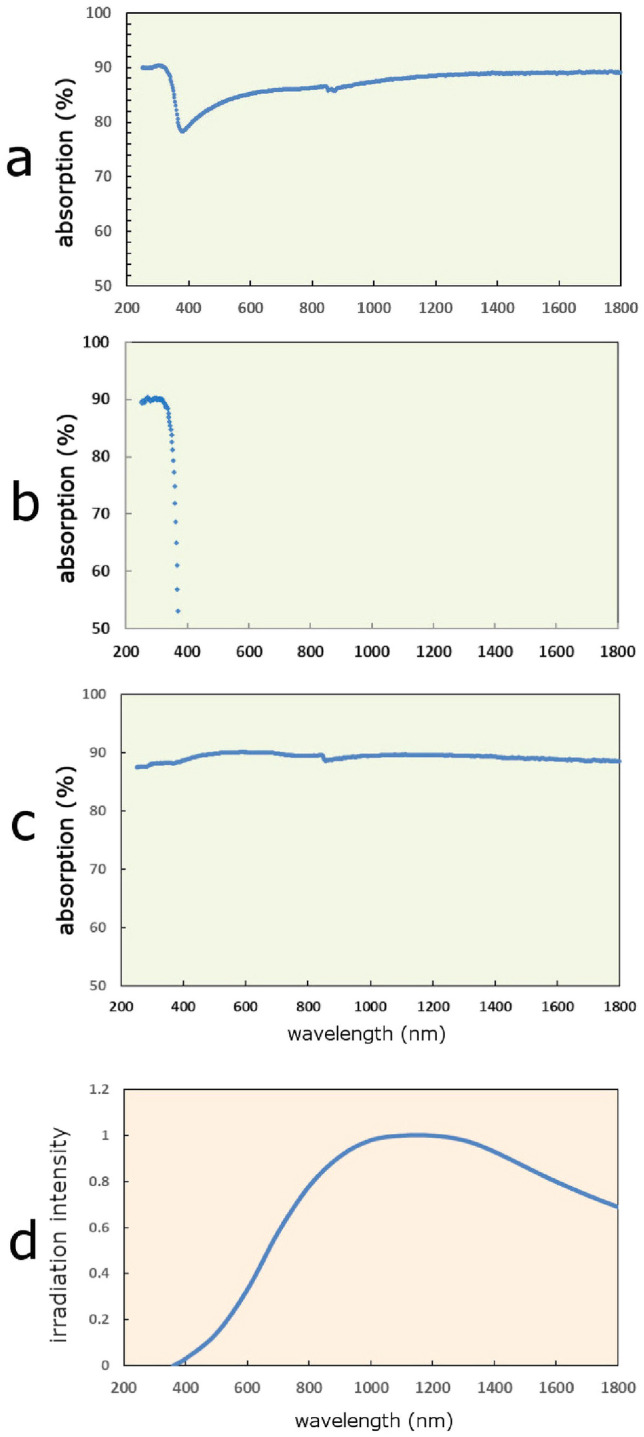
Absorbing curve or irradiation curve as a function of wavelength of elements that were used in this report. (**a**) Absorbing curve as a function of wavelength of TiO_2_/ Fe_3_O_4_ (1/1). (**b**) Absorbing curve as a function of wavelength of TiO_2_. (**c**) Absorbing curve as a function of wavelength of Fe_3_O_4_. (**d**) Irradiation intensity curve of 2700K black body radiation corresponding to radiation of typical tungsten light.

Figure 4 shows absorbing curve (measured by JASCO Corporation Spectrophotometer V570) or irradiation curve as a function of wavelength regarding elements that were used in this report. TiO_2_/Fe_3_O_4_ (1/1) composite can absorb rays of 200–1800 nm in wavelength (Fig. 4a). Rays of less than 380 nm in wavelength can be absorbed by TiO_2_ component (Fig. 4b), and rays of more than 400 nm in wavelength can be absorbed by Fe_3_O_4_ component (Fig. 4c). As irradiation area of both 365 nm LED light and tungsten light (Fig. 4d) covers 360–1800 nm in wavelength, system using both 365 nm LED light and tungsten light have good affinity with TiO_2_/Fe_3_O_4_ (1/1) composite. And irradiation with both 365 nm LED light and tungsten light is good alternative to that of solar light. Moreover, the irradiation system was very useful for experiments at night or on rainy day.

Solubility of N_2_ into water is 0.024 at 0 °C,0.016 at 20 °C, 0.012 cm^3^/H_2_Ocm^3^ at 40 °C, respectively, and solubility of CO_2_ into water is 1.71 at 0 °C, 0.88 at 20 °C and 0.53 cm^3^/H_2_Ocm^3^ at 40 °C respectively^49^. As solubility of N_2_ molecules in air into water is very small than that of CO_2_ into water, N_2_ molecules cannot reach surface of composites covered with thin water layer, Thus N_2_ molecules in air cannot become N_2_ source for ammonia synthesis after generation of thin water layer. While, N_2_ molecules that adsorbed on the surface of the composites before formation of thin water layer can become N_2_ source. Moreover, as adsorbing N_2_ molecules are protected from going away to atmosphere by thin water layer, they can be efficiently used for ammonia generation.

In the experimental No. 33 (experiment using Ar gas), author noticed that “adsorption of N_2_ molecules in air onto surface of composites” was very important. Moreover, author hit upon BET method for surface-area measurement. In the method, N_2_ molecules adsorb onto surface of object that was held in atmosphere cooled to liquid N_2_ temperature (−196 °C). And, as nm-sized TiO_2_ particles have very large surface area, adsorbing N_2_ molecules would be large quantity at liquid N_2_ temperature. However holding in freezer (approximately −20 °C) that is easily available method than holding in liquid N_2_ temperature was adopted, although holding in liquid N_2_ temperature may provide larger ammonia yield.

Graeme et al. reported importance of interface^50^. Endoh et al. reported that composites prepared though “ground in mortar” of metal oxides provided larger ammonia yield in tables in their reports^5,6^, that is, they suggested the effect of interface. This time, from experimental No. 10–13, effect of interface was revealed. Thus, the interface provided effect of binding both photocatalytic reaction of TiO_2_ and catalytic one of Fe_3_O_4_.

With regard to practical use, this method cannot operate continuously, because the water layer on the composite evaporates and disappear within short time. However, it can operate quasi-continuously by repeats of cooling in refrigerator (or freezer) and irradiation of various lights.

And the thin water layer over composites played following roles,

1. Preventing oxidation of synthesised ammonia on surface of composites (Effect of wet condition).
2. Splitting itself to generates H^+^ ions and electrons through photocatalytic reaction.
3. Preventing N_2_ molecules adsorbed on surface of composites to go away into atmosphere before or during irradiation of light.

From results and discussion, possible macro mechanism is following:

1. N_2_ molecules in air adsorb onto surface of TiO_2_/Fe_3_O_4_ composite during holding of test unite in refrigerator (or freezer).
2. Physical adsorbing N_2_ molecules move to interface of both TiO_2_ particle and Fe_3_O_4_ one.
3. N_2_ molecules physically adsorbed change to dissociated adsorption at the interface or on Fe_3_O_4_ surface in the neighbourhood of the interface.
4. Catalytic reducing reaction of dissociative adsorbing N-atom at the interface or on Fe_3_O_4_ surface in the neighbourhood of the interface occurs.
  In the case using solar light or 365nm LED light, high energy electrons (e^-)^ generated by TiO_2_ photocatalytic effect strongly accelerate the catalytic reducing reaction.
5. Desorption of ammonia generated from the interface into water layer.
6. Evaporation of ammonia generated from the water layer into air.
7. Repeating of 2–6 until the thin water layer evaporate and disappear.

## Conclusion

The WLOC method with TiO_2_/Fe_3_O_4_ (1/1) composite for ammonia generation from N_2_ molecules in air under ambient condition and under irradiation of various lights provided successful results. N_2_ molecules adsorbed onto composite’s surface during held in refrigerator or freezer. Holding in freezer generated large quantity of ammonia. TiO_2_/ Fe_3_O_4_ (1/1) composite produced interface consisted of TiO_2_ particles and Fe_3_O_4_ ones, and interface offered catalytic reaction sites. Adsorbing N_2_ molecules onto surface of mainly nm-size TiO_2_ component were reduced into ammonia under ambient condition. In the case using soler light or 365 nm LED light, the reaction was catalytic reaction promoted by photocatalytic one. Solar light as light source was useful, and using both 365 nm LED light and tungsten light also was effective, even using only tungsten light was effective. Author’s WLOC method was very effective for reducing stable molecules like N_2_ under ambient condition. Moreover, this method is considered to be suitable for small scale ammonia production.

## Methods

### Preparation of the composites (c)

In the case of TiO_2_/Fe_3_O_4_ = 1/1, procedure is following.

First, 0.5 g of nanometre-sized anatase TiO_2_ particles (a) (Kanto Kagaku Co. Ltd., Titanium (IV) oxide, powder, Anatase, Cat. No. 49526-76, IOLITEK Ionic Liquids Technologies GmbH, Titanium (IV) oxide powder, particle size: 10–30 nm purity: 99.5% (NO-0058-HP-0025) Lot: TNO058011 and 0.5 g of reagent-grade Fe_3_O_4_ particles (b) (Kanto Kagaku Co. Ltd., Iron Oxide, Black Assay: min 95.0%, Cat. No. 20073- 01) were placed into a small ceramic pot and mixed uniformly. They were then forcefully pressed and agglomerated at room temperature to form the composites (c).

In the case of experimental No. 42–43, ZrO_2_Ep (ITEC Co. Ltd., particle size; 6 nm) and ZrO_2_3N (Kanto Kagaku Co. Ltd., purity: 99.9% particle size: 10 μm) were used.

## Experimental procedure

Next, 0.2 g of the composites (c) was scattered uniformly onto a copper plate, which was placed in a glass laboratory dish (inner diameter: 56 mm). The assembly with (c) scattered on the Cu plate in a glass laboratory dish is referred to as the test unit.

Initially, particles or molecules adsorbed onto the composites surface were removed by eliminating static electricity using an ionizer (Kasuga Denki. Inc., Model KD-150W). The treatment time was 1 h. After the test unit was kept for more than 24 h (desirably 48 h) in a refrigerator (inner temperature of the refrigerator: 1–5 °C), it was taken out and placed into a transparent gas-barrier plastic bag (Okura Kogyo Co. Ltd., OE-4, 200 × 300 mm^2^). Thermometer was also placed into the gas-barrier plastic bag. The bag was heat-sealed at its entrance. Urethane tape (1 × 1 cm^2^) was adhered to the outside surface of the bag. After the air in the bag was removed using a plastic syringe, new air was injected into the bag. The needle of the syringe was inserted though the urethane tape to avoid leaving a small hole after the needle was removed. The inner volume of the bag was 1000 ml. The bag was then placed on the ground facing the sun or placed below artificial lights. The injected air was at a sufficient temperature and humidity. With the using of 0.2 g of the composites (c), the desired temperature and humidity were approximately 30 °C and 60–90%, respectively. After irradiation by real solar light or lights of both 365nmLED light and tungsten light, 365 nm LED light only or tungsten light only through the bag for 5 min., temperature in bag was cooled down to less than 35 °C, then the concentrations (ppm) of ammonia was measured by a gas-detecting tube [Gastec Co. Ltd., tube No. 3L (measuring range: 0.5–1 ppm, 1–30 ppm, and 30–78 ppm; three measuring ranges are available by altering the suction volume) and Tube No. 3La (measuring range: 2.5–5 ppm and 5–100 ppm and 100–220 ppm); three measuring ranges are available by altering the suction volume)]. The tube was inserted thorough the plastic tape adhered to the outside surface of the bag and the concentrations were measured; after the tubes were removed, the remaining hole was immediately sealed with another piece of tape. The inner volume of the bag was measured by extracting the gas with the plastic syringe. The measured concentrations (ppm) of ammonia was then corrected for temperature and expressed in terms of μmol/g:

$${\text{Micromole}}/{\text{g}}\, = \,{\text{acV}}/({22},{4}00\, \times \,{\text{g}})$$

where a is temperature-correction-factor (0.86 or 0.93), c is ammonia concentration (ppm), V is the inner volume (ml) after irradiation, and g is the mass of composite (c) used (g).

The intensity of the UV light (mW/cm^2^) with a wavelength of 365 nm was measured using a Unimeter (Ushio Co. Ltd., UIT-201 and UVD-365PD) during the irradiation of solar light during each experiment; the measurements were performed near the point where the composites were placed in the bag. The intensity was measured every 1 min. In the case of using 365 nm LED light and tungsten light, 365 nm LED light was constantly set at the height offered intensity of 1.5 mW/cm^2^ over composite. Tungsten light was ordinarily set at the point that distance between composite and light was approximately 30 cm.

## Data Availability

All data generated or analysed during this study are included in this published article.
